# Pathogenicity of SARS‐CoV‐2 Omicron

**DOI:** 10.1002/ctm2.880

**Published:** 2022-05-11

**Authors:** Hin Chu, Kwok‐Yung Yuen

**Affiliations:** ^1^ State Key Laboratory of Emerging Infectious Diseases Li Ka Shing Faculty of Medicine Department of Microbiology Carol Yu Centre for Infection School of Clinical Medicine The University of Hong Kong Hong Kong Special Administrative Region Pokfulam China; ^2^ Department of Clinical Microbiology and Infection Control The University of Hong Kong‐Shenzhen Hospital Shenzhen Guangdong Province China

The Coronavirus Disease 2019 (COVID‐19) pandemic caused by severe acute respiratory syndrome coronavirus 2 (SARS‐CoV‐2) continues to affect many parts of the world more than 2 years since the pandemic started.^1, 2^ As of 23 April 2022, SARS‐CoV‐2 has infected over 508 million people with more than 6.2 million deaths. As SARS‐CoV‐2 continues to spread among humans, new variants with mutations that may modulate viral transmissibility, pathogenicity and antibody evasion continue to emerge. Currently, the World Health Organization (WHO) has identified five SARS‐CoV‐2 variants as Variant of Concern (VOC), including Alpha (B.1.1.7), Beta (B.1.351), Gamma (P.1), Delta (B.1.617.2) and Omicron (B.1.529).

Omicron, the most recently defined VOC, was first reported in November 2021 in South Africa. Omicron demonstrated robust transmissibility among the human population and has quickly replaced Delta as the dominant circulating SARS‐CoV‐2 variant.^3^ Genetically, Omicron contains a large number of mutations in the spike protein, including 30 amino acid substitutions, three short deletions and one insertion, compared with the ancestral SARS‐CoV‐2. This unusually high number of mutations gives Omicron the ability to efficiently escape from neutralising antibody in convalescent or vaccinated sera, and modify its capacity in cell entry, replication and pathogenesis.

## 1 ATTENUATED REPLICATION AND PATHOGENICITY OF OMICRON

To investigate the pathogenicity of Omicron, we infected K18‐human angiotensin‐converting enzyme 2 (hACE2) mice with SARS‐CoV‐2 wild‐type (WT), Alpha, Beta, Delta and Omicron using the same virus inoculum.^4^ We found that Omicron infection resulted in the least body weight body loss and the highest survival rate in the infected mice among all evaluated virus strains.^4^ In keeping with these clinical observations, virological assessments of mouse tissue samples revealed that virus replication and virus‐induced lung damage were both significantly reduced in Omicron‐infected mice in comparison to WT‐ or Delta‐infected K18‐hACE2 mice. Since Omicron contains the N501Y substitution in its spike protein that allows it to infect WT mice,^5^ we compared the replication of Omicron and the N501Y‐carrying Alpha in C57B6 WT mice. Our results showed that the replication of Omicron was significantly attenuated in the respiratory tract in comparison to that of Alpha.^4^ Together, these findings indicate that Omicron is attenuated compared with SARS‐CoV‐2 WT and previous VOCs,^4^ which are in keeping with the results from Syrian hamster studies^6^, ^7^, ^8^, ^9^ and more recently from clinical studies which demonstrated the generally lower disease severity of Omicron than other SARS‐CoV‐2 strains.^10^, ^11^, ^12^, ^13^, ^14^

## 2 MECHANISM BEHIND THE ATTENUATED PATHOGENICITY

Mechanistically, we showed that Omicron is deficient in spike cleavage, leading to inefficient transmembrane protease, serine 2 (TMPRSS2) usage.^4^ Since SARS‐CoV‐2 enters lung cells primarily through the TMPRSS2‐mediated plasma membrane entry pathway,^15^ the inefficient spike cleavage and TMPRSS2 usage results in significantly attenuated virus replication in lungs and dramatically reduces virus pathogenicity. This finding has major implications on the potential treatment strategy for Omicron as it is less susceptible than ancestral SARS‐CoV‐2 to TMPRSS2 inhibitors such as camostat mesylate.

## 3 FUTURE PERSPECTIVES

The findings from our study suggest that compared with the ancestral WT SARS‐CoV‐2 or previous VOCs, different clinical treatment strategies and public health control measures should be implemented for the optimal control of the current COVID‐19 pandemic caused by the Omicron wave. Furthermore, continuous surveillance revealed different sublineages of Omicron in addition to BA.1, including BA.1.1, BA.2, BA.3, BA.4 and BA.5. While studies from us and others revealed the attenuated pathogenicity of Omicron BA.1, the pathogenicity of the other Omicron sublineages remain largely unexplored. This is particularly important since BA.2 exhibits even higher transmissibility than BA.1^16^ and has now replaced BA.1 and BA.1.1 as the dominant circulating SARS‐CoV‐2 variant. In addition, recombination variant between Omicron BA.1 and BA.2, known as XE, as well as recombination variants between Omicron BA.1 and Delta, known as XD and XF, have recently been reported.^17^ The virological characteristics of these new SARS‐CoV‐2 variants should be further investigated. The knowledge obtained will be highly important for setting a balanced and optimal public health control measure for the ongoing COVID‐19 pandemic.

If we learn from the past history of the four mild common cold coronaviruses, we should vaccinate as much as possible to prevent severe diseases, and then allow SARS‐CoV‐2 to circulate during the summer at a low level so that our population immunity can be continuously boosted naturally by the milder Omicron variant. The border and social distancing measures should be relaxed in a gradual manner. When winter comes or another variant emerges, the elderly and chronically sick should receive another booster dose of the most updated coronavirus vaccine together with the seasonal flu vaccination to boost their immunity without resorting to border control and social distancing again. As time goes by, our whole population immunity against severe disease would be consolidated by the continuous circulation of mild SARS‐CoV‐2 variants. Finally, SARS‐CoV‐2 will become just one of these common cold coronavirus causing mild seasonal outbreaks. Note that if we stop low level circulation by elimination measures of mass testing, isolation of all cases found by compulsory universal testing and quarantine of all contacts, we may never build up sufficient natural immunity after paying a huge psychosocial and economic price. Nature can be very unforgiving and our elderly population and patients with chronic diseases may be severely impacted during another wave of COVID‐19.

## CONFLICT OF INTEREST

The authors declare no conflict of interest.
